# Dietary Salt Intake and Discretionary Salt Use in Two General Population Samples in Australia: 2011 and 2014

**DOI:** 10.3390/nu7125545

**Published:** 2015-12-16

**Authors:** Caryl Nowson, Karen Lim, Carley Grimes, Siobhan O’Halloran, Mary Anne Land, Jacqui Webster, Jonathan Shaw, John Chalmers, Wayne Smith, Victoria Flood, Mark Woodward, Bruce Neal

**Affiliations:** 1 School of Exercise and Nutrition Sciences, Deakin University, Centre for Physical Activity and Nutrition Research, Locked Bag 20000, Waurn Ponds, Geelong VIC 3220 Melbourne, Australia; k.lim@deakin.edu.au (K.L.); carley.grimes@deakin.edu.au (C.G.); s.ohalloran@deakin.edu.au (S.O.); 2 George Institute for Global Health, University of Sydney and Royal Prince Alfred Hospital, Sydney 2050, Australia; maland@georgeinstitute.org.au (M.A.L.); chalmers@georgeinstitute.org.au (J.C.); markw@georgeinstitute.org.au (M.W.); bneal@georgeinstitute.org.au (B.N.); 3 WHO Collaborating Centre on Population Salt Reduction, George Institute for Global Health, University of Sydney, Sydney 2050, Australia; jwebster@georgeinstitute.org.au; 4 Clinical Diabetes and Epidemiology, Baker IDI Heart & Diabetes Institute, Melbourne 3004, Australia; Jonathan.Shaw@bakeridi.edu.au; 5 Environmental Health Branch, New South Wales Health, Sydney 2059, Australia; wsmit@doh.health.nsw.gov.au; 6 Faculty of Health Sciences, University of Sydney, Sydney 2141, Australia; vicki.flood@sydney.edu.au; 7 St Vincent’s Health Network, Sydney, 2010, Australia; 8 Medical Sciences Division, University of Oxford, Oxford OX1 3BD, UK; 9 Sydney Medical School, University of Sydney, Sydney 2050, Australia; 10 School of Public Health, Faculty of Medicine, Imperial College London, London SW7 2AZ, UK

**Keywords:** salt, sodium, sodium chloride, diet, regional, urinary sodium, Australia

## Abstract

The limited Australian measures to reduce population sodium intake through national initiatives targeting sodium in the food supply have not been evaluated. The aim was, thus, to assess if there has been a change in salt intake and discretionary salt use between 2011 and 2014 in the state of Victoria, Australia. Adults drawn from a population sample provided 24 h urine collections and reported discretionary salt use in 2011 and 2014. The final sample included 307 subjects who participated in both surveys, 291 who participated in 2011 only, and 135 subjects who participated in 2014 only. Analysis included adjustment for age, gender, metropolitan area, weekend collection and participation in both surveys, where appropriate. In 2011, 598 participants: 53% female, age 57.1(12.0)(SD) years and in 2014, 442 participants: 53% female, age 61.2(10.7) years provided valid urine collections, with no difference in the mean urinary salt excretion between 2011: 7.9 (7.6, 8.2) (95% CI) g/salt/day and 2014: 7.8 (7.5, 8.1) g/salt/day (*p* = 0.589), and no difference in discretionary salt use: 35% (2011) and 36% (2014) reported adding salt sometimes or often/always at the table (*p* = 0.76). Those that sometimes or often/always added salt at the table and when cooking had 0.7 (0.7, 0.8) g/salt/day (*p* = 0.0016) higher salt excretion. There is no indication over this 3-year period that national salt reduction initiatives targeting the food supply have resulted in a population reduction in salt intake. More concerted efforts are required to reduce the salt content of manufactured foods, together with a consumer education campaign targeting the use of discretionary salt.

## 1. Introduction

Reducing average salt intake from current high levels (8–9 g/salt/day) in Australia towards the World Health Organization’s (WHO) recommended upper daily limit of 5 g per person could significantly reduce cardiovascular events and mortality [1]. It has been estimated that reducing the sodium content of processed foods by even a modest 15% could prevent 5800 myocardial infarctions and 4900 strokes in Australia and New Zealand over a ten-year period, preventing over 2000 deaths [2].

In Australia, studies using the objective measure of 24 h urine collections to assess daily salt intake have generally been conducted on convenience samples of adults. Studies published between 2003 and 2011 indicate average intakes of between 7.0 g/salt/day for women and 9.6 g/salt/day for men (expressed as grams of salt: mmol sodium × 23 × 2.54/1000) [3,4,5,6,7,8,9,10,11,12]. The most recent study, in 419 participants (55% females) drawn from the population of Lithgow—a regional town in New South Wales—in 2011, found the mean dietary salt intake to be 8.8 g/day with men excreting 10.3(3.8) (SD) g/salt/day and women 7.6(3) g/salt/day [3].

The primary determinant of dietary salt intake in developed countries is manufactured foods, which has been estimated to contribute more than 75% of the daily amount of salt consumed [13,14]. Discretionary salt accounts for a smaller contribution of 10%–15% of daily salt intake [15,16]. Estimations of dietary salt intake utilizing dietary recall methods indicate a lower daily intake to that found in 24 h urine collections, ranging from an approximate average daily salt intake of 6 g/day for women and 8 g/day for men [4,5,6,17,18]. Although the 2011–2013 National Nutrition and Physical Activity Survey indicates a lower average intake of 6.9 g/salt/day for males and 5.2 g/salt/day for females [19] from previous surveys, these dietary assessments do not include an estimate of discretionary salt use and rely on generic food composition data making estimation of population change in total salt intake problematic.

One of the key strategies to address the high sodium content of manufactured foods in Australia was the establishment of the Food and Health Dialogue (FHD) in 2009. As part of the FHD, a Reformulation Working Group was established to investigate priority food categories, which have been identified as presenting an opportunity for public health benefit through reformulation and where appropriate, portion sizing and consumer messaging targets. This initiative aimed to encourage food manufacturers to voluntary reduce the amount of salt added to processed foods, by setting sodium reduction targets for specific food categories. A recent review of the impact of this initiative, between 2010 and 2013 concluded that although the goals of the FHD were laudable, there were no indicators of the extent to which population exposure to target nutrients—which included sodium—had changed, nor any positive or negative health impacts on the population [20]. Therefore we aimed to assess daily dietary salt intake, using 24 h urine collections, together with reported discretionary salt use, in a sample of adults in the state of Victoria, at two time points: 2011 and 2014 and assess if there was a change in salt intake during this period.

## 2. Methods

### 2.1. 2011 Survey

Study participants were recruited from a sample of 3653 people (38% response rate) participating in the Victorian Health Monitor survey (VHM) (May 2009 to April 2010) [21]. This statewide survey included non-institutionalized adults aged 18–75 years residing in private dwellings in 50 randomly selected metropolitan and rural areas, based on a stratified cluster sample within the eight Department of Health regions in Victoria. The final sample included 25 Census Collection Districts (CDs) from the metropolitan area and 25 CDs from rural areas of Victoria. In 2011, 3487 people who participated in the VHM survey, and lived within 100 km of a commercial pathology center were invited to participate by providing a 24 h urine collection. Participants who provided a urine collection also completed a telephone-administered interview.

It was estimated that a sample size of 612 would be sufficient to describe a mean intake of urinary sodium within a 95% confidence interval of 5 mmol/day, assuming a standard deviation of 63 mmol/day [7]. Making an allowance for non-participation and incomplete samples we therefore followed up 697 of the 1003 participants who indicated interest in participating from the 3487 adults invited from the VHM survey. In this sample of 1003 participants we followed up all of those at the youngest end of the age distribution range to maximize the number of participants less than 40 years of age, where the response rate was lower.

### 2.2. 2014 Survey

The 2014 survey comprised of people who participated in the 2011 survey who indicated their willingness to be approached in a follow up study, together with those who had previously indicated their willingness to participate in the 2011 study, but were not included (*n* = 306) because we had reached our specified quota. These 306 participants who had indicated their willingness to participate previously, but were not included, were sent a letter in 2014 requesting participation in the follow-up study. Participation involved collection of a 24 h urine sample, completion of a questionnaire assessing discretionary salt use and knowledge and attitudes to salt intake, and reported information on body weight, which was returned by mail.

All participants provided informed consent and the studies were approved by the ethics committees of the Alfred Hospital (Project No. 421/10) and Deakin University (Project No. 2011-025).

### 2.3. 24 h Urine Collections

Participants indicating their interest in participating received a phone call from researchers who explained the process. Participants were sent urine collection bottles in the mail, with detailed pictorial instructions for the collection, together with a recording sheet to note start and finish times of their 24 h collections. Researchers identified local pathology centers for each participant and participants deposited their 24 h urine collection at their local pathology center. The pathology service then sent the sample for analysis to their main laboratory. Participants were advised to drop off the 24 h collection as soon as possible after completion. Participants recorded the start and end times for their urine collection and reported any missed urine during the 24 h collection period. Within the 24 h collection period subjects collected a spot or timed urine collection into a separate bottle which was analyzed separately and the results added to the other collection to obtain the total excretion for 24 h. The collection of spot urines within the 24 h urine collection period formed part of a separate validation study. The reported start and finish times of the collections were used to calculate total daily excretion. The length of collection times for valid urine collections varied from 14 to 31 h with all urinary results standardized to a 24 h period. It is known that 24 h creatinine excretion in adults can be predicted from body weight, height, age and gender and has been used in a number of studies to assess completeness of urine collections [22]. In addition, criteria based on low urine volume, reported missed collections and extreme statistical outliers, which are similar to those previously utilized were used to identify probable over and under collection of 24 h samples [3].

The urine samples were received, processed and analyzed by the Australian accredited pathology service centers of Healthscope and Dorevitch. Urinary sodium and potassium concentration were determined using ion-selective electrodes and urinary creatinine concentration was determined from the kinetic Jaffe reaction using the Advia 2400 Clinical Chemistry System (Siemens).

### 2.4. Demographic, Anthropometric and Dietary Data

Demographic data and height measurements were obtained as part of the VHM survey. Height was measured to the nearest 0.1 cm without shoes using a stadiometer. Annual household income and highest level of education of the participant were self-reported in VHM and used as indicators of socioeconomic status. In 2011 participants provided information on their current body weight together with information on salt use during cooking and at the table during a phone interview which occurred within three days of the 24-h urine collection. There were 5 response options for use of salt in cooking and at the table (never, rarely, sometimes, often, always). Body mass index (weight kg/height m^2^) was calculated from self-reported weight at the time of interview in 2011 and self-reported weight on return of the questionnaire for 2014.

All participants received a shopping voucher to the value of $20, or a double-pass movie ticket on completion of each 24 h urine collection (2011, 2014) together with a summary of their individual results.

### 2.5. Statistical Analysis

Participant characteristics are presented as mean (SD), mean (95% CI) or *n* (%). Baseline comparisons were made using t-tests and chi-square tests for continuous and categorical variables, respectively. Linear mixed effect modelling with maximum likelihood estimation was applied to assess the change in sodium intakes from 2011 to 2014, adjusting for demographic and anthropometric covariates (*i.e.*, fixed effects: gender, age, self-reported body weight, urban/regional residence, weekend day collections). Random effects were included to account for variance within participants and within recruitment clusters (*i.e.*, cluster 1 participated in 2011 only, cluster 2 participated in 2014 only, cluster 3 participated in both 2011 and 2014). This same model was used to obtain overall and sex-specific adjusted mean 24-h sodium excretion (mmol/24 h) and salt equivalents (salt (g) (mmol sodium × 23 × 2.54/1000). For descriptive purposes and to assess associations between discretionary salt use and sodium excretion, the five response options were contracted into two categories: never or rarely; and sometimes, often, or always. Assessment of the association of discretionary salt use with 24 h sodium excretion was adjusted for gender using ordinary least squares linear regression and these adjusted values were contrasted across the two categories of discretionary salt use (Never or Rarely, and Sometimes, Often or Always) with post estimation procedures (standard errors derived using delta method). We derived odds ratios using binary logistic regression to assess associations with discretionary salt use. Residential postcodes were used to classify place of residence as either city/metropolitan (Melbourne metropolitan) or regional/rural [23]. In addition the state based (*i.e.*, Victoria) Index of Relative Socio-Economic Disadvantage (SEIFA) was derived from residential postcodes [24]. Data were analyzed using Stata/SE 13.1 (StataCorp, College Station, TX, USA).

## 3. Results

In 2011, of the 697 formally invited to participate in the study, 605 subjects participated. In 2014, 594 of those participating in 2011 were approached, together with an additional 306 people who expressed interest in the 2011 survey but did not participate resulting a total sample of 448 in 2014 of which 315 had previously participated and 133 participated for the first time.

Seven urine collections in 2011 and 6 collections in 2014 were excluded due to likely errors in collection: women with 24-h creatinine <4 mmol/day (*n* = 3), men with 24-h creatinine <6 mmol (*n* = 2), men with 24 h creatinine >3 SD from mean (*n* = 3), women with 24 h creatinine >3 SD from mean (*n* = 2), 24 h urine volume <500 mL (*n* = 2), and one reported missing more than one void within the collection period.

The final sample of valid urines included 307 subjects who participated in both surveys (2011 and 2014) (*n* = 614 urine collections), 291 from those who participated in 2011 only, and 135 subjects who participated for the first time in 2014. Seven individuals who participated in both years had invalid urine collections in one year (4 in 2011, 3 in 2014). Therefore there were 598 valid urines provided in 2011 and 442 in 2014, with an overall total of 1040 urine collections from 733 individuals. Overall there was a balanced gender split, approximately 53% females and no difference in gender between the two years of collections (Table 1). Men excreted more salt than women by 37%: 9.2 (8.9, 9.5) (mean (95%CI)) g/salt/day *versus* 6.7 (6.4, 7.0) g/salt/day; *p* < 0.001, and this difference remained 23% higher even after adjustment for body weight: 8.7 (8.4, 9.0) g/salt/day *vs.* 7.1 (6.8, 7.4) g/salt/day; *p* < 0.001 (combined 2011 and 2014). The 2014 group was older by about 4 years, which reflects the 3 years time difference between surveys. Although 18% more of the 2014 group had completed tertiary education compared to the 2011 group (*p* = 0.018), annual household income did not differ between the years (*p* = 0.4).

**Table 1:** Demographic characteristics (2011, 2014).

Mean (95% Confidence Intervals)			
	2011 (*n* = 598)	2014 (*n* = 442)	2011 & 2014 Combined (*n* = 1040)
Women, *n* (%)	320 (53.5%)	235 (53.2%)	555 (53.4%)
Age (year)	57.1 (56.2, 58.1)	61.2 (60.2, 62.2) ^a^	58.9 (58.2, 59.6)
Weight (kg) ^f^	76.2 (75.0, 77.5)	75.5 (74.0, 77.0)	75.9 (75.0, 76.9)
Body Mass Index (kg/m^2^) ^f^	26.6 (26.2, 27.0)	26.4 (25.9, 26.8)	26.5 (26.2, 26.8)
Urinary volume (mL/day)	2085 (2056, 2154)	2052 (1974, 2130)	2071 (2019, 2123)
Creatinine (mmol/day)	11.7 (11.5, 12.0)	11.5 (11.2, 11.9)	11.6 (11.4, 11.9)
Urinary sodium (mmol/day)	137.9 (133.4, 142.5)	130.1 (124.6, 135.7) ^e^	134.6 (131.1, 138.1)
Urinary salt (g/day) ^g^	8.1 (7.8, 8.3)	7.6 (7.3, 7.9) ^e^	7.9 (7.7, 8.1)
Urinary salt (g/day) (adjusted) ^h^	8.0 (7.7, 8.2)	7.8 (7.5, 8.1)	7.9 (7.7, 8.1)
Urinary salt (g/day) (adjusted) ^i^	7.9 (7.6, 8.2)	7.8 (7.5, 8.1)	7.8 (7.7, 8.1)
Urinary potassium (mmol/day)	77.8 (75.8, 79.7)	76.5 (74.1, 78.9)	77.2 (75.7, 78.7)
Urinary Na^+^/K^+^ ratio (/day)	1.9 (1.8, 1.9)	1.8 (1.7, 1.9)	1.8 (1.8, 1.9)
(*n*, frequency) (%)			
Weekend urine sample	179 (29.9%)	99 (22.4%) ^b^	278 (26.7%)
Metropolitan Melbourne	366 (61.2%)	241 (54.5%) ^c^	607 (58.4%)
Age range			
<40 years	65 (10.9%)	16 (3.6%) ^d^	81 (7.8%)
40–60 years	261 (43.7%)	165 (37.3%) ^d^	426 (41%)
≥60 years	272 (45.5%)	261 (59.1%) ^d^	533 (51.3%)
Annual household income			
≤$40,000	156 (26.1%)	99 (22.4%)	255 (24.5%)
$40–70,000	144 (24.1%)	102 (23.1%)	246 (23.7%)
>$70,000	265 (44.3%)	218 (49.3%)	483 (46.4%)
Don’t know/Refused	33 (5.5%)	23 (5.2%)	56 (5.4%)
Highest level of education			
No tertiary education	346 (57.9%)	223 (50.5%) ^e^	569 (54.7%)
With tertiary education	252 (42.1%)	219 (49.6%) ^e^	471 (45.3%)
SEIFA IQR (Vic) ^i^			
<25th percentile	70 (11.7%)	50 (11.3%)	120 (11.5%)
25–75th percentile	262 (43.8%)	180 (40.7%)	442 (42.5%)
≥75th percentile	266 (44.5%)	212 (48%)	478 (46%)

^a^ *p* < 0.001 *T*-test; ^b^ <0.01 chi squared (χ^2^); ^c^ <0.05 chi squared (χ^2^); ^d^ <0.001 chi squared (χ^2^); ^e^ <0.05 *T*-test; ^f^ self-reported weight; ^g^ expressed as grams of salt (mmol sodium × 23 × 2.54/1000); ^h^ adjusted for age, gender, greater Melbourne area, weekend/weekday collection; ^i^ adjusted for age, gender, greater Melbourne area, weekend/weekday, participation in both surveys.

The sample in 2014 also had a slightly lower proportion of residents (7%) from the greater Melbourne area and 7% fewer performed their urine collections on a weekend day in 2014. Salt excretion was higher on weekend days (*i.e.*, Saturday and Sunday) than week days: 8.4 (8.0, 8.8) g/salt/day *versus* 7.7 (7.4, 8.0) g/salt/day; *p* = 0.003.

Daily urinary salt excretion was higher in residents of the greater Melbourne area compared to regional Victoria: 8.1 (7.8, 8.4) g/salt/day *vs.* 7.6 (7.3, 8.0); *p* = 0.054. This regional difference remained after adjustment for age, gender, day of collection, tertiary education, and participation in both studies: 8.1 (7.8, 8.3) *versus* 7.6 (7.2, 7.9) g/salt/day; *p* = 0.02). When adjusting for age, gender, regional/metro & participation in both years, those with tertiary education had lower salt excretion 7.4 (7.1, 7.7) g/salt/day *versus* 8.2 (7.9, 8.5) g/salt/day for participants with no tertiary education (*p* < 0.001). Furthermore the participants in the highest income classification (>$70 K) excreted less salt compared to the lowest income classification (<40 K), 7.6 (7.3, 7.9) g/salt/day and 8.4 (8.0, 8.8) g/salt/day respectively (*p* = 0.004). Postcode SEIFA index did not predict 24 h salt excretion (data not shown).

The 2014 sample had a slightly lower mean 24 h excretion of salt, however once adjusted for the key confounders, age, gender, metropolitan region, weekend collection and participation in both surveys, there was no difference between sampling years mean salt excretion: 7.9 g/salt/day 2011 *versus* 7.8 g/salt/day in 2014; *p* = 0.63. There was no difference between years when split by gender (*p* = 0.59): Males in 2011 (*n* = 278) 9.2 (8.9, 9.6) g/salt/day and in 2014 (*n* = 207) 9.1 (8.7, 9.5) g/salt/day, and females in 2011 (*n* = 320) 6.8 (6.4, 7.1) g/salt/day and in 2014 (*n* = 235) 6.6 (6.3, 7.0) g/salt/day (adjusted for age, metropolitan region, weekend collection and participation in both surveys).

### Use of Discretionary Salt

**Figure 1:**
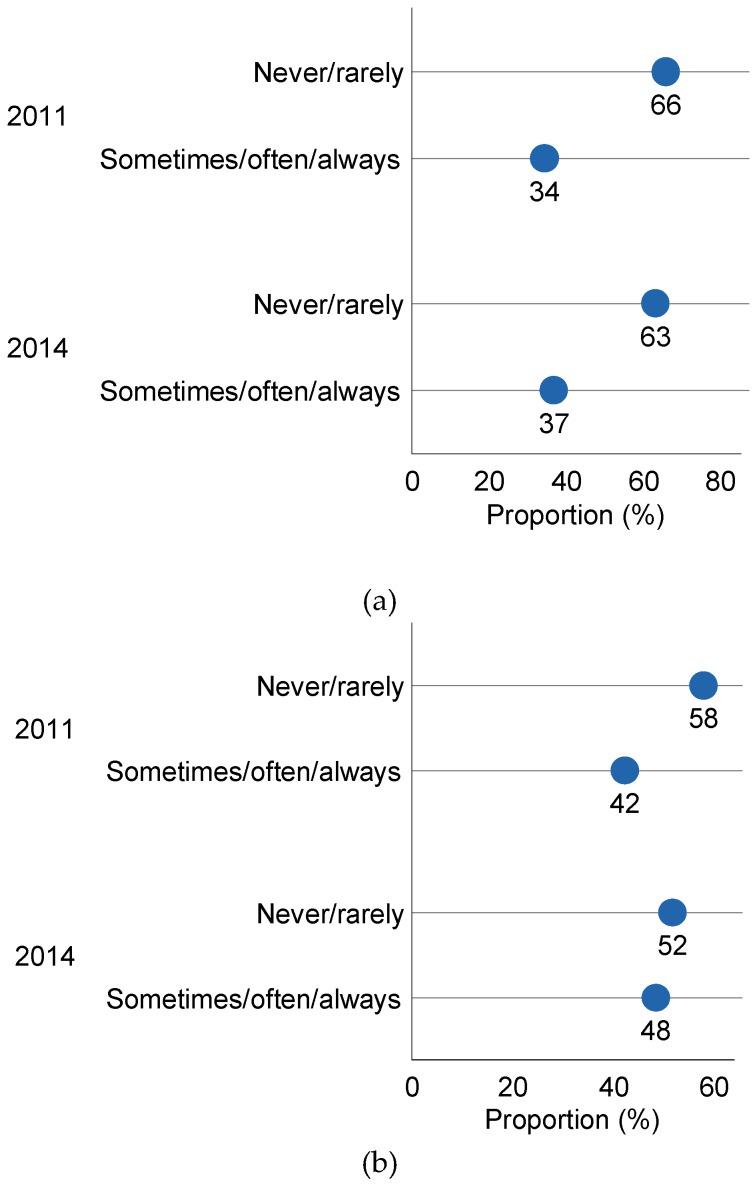
Frequency of salt use at the table and in cooking in 2011 and 2014. (**a**) Reported use of table salt in 2011 (*n* = 598) and 2014 (*n* = 442); (**b**) Reported use of cooking salt in 2011 (*n* = 580 ^a^) and 2014 (*n* = 438 ^b^); ^a^ Excludes 2 females and 16 males who reported not cooking; ^b^ Excludes 4 males who reported not cooking.

The reported frequency of use of salt at the table and in the cooking did not differ between 2011 and 2014 (Figure 1) such that 35% in 2011 and 36% in 2014 reporting adding salt sometimes or often/always at the table (*p* = 0.76), and 44% in 2011 and 47% in 2014 reporting adding salt sometimes or often/always during cooking (*p* = 0.29) (gender adjusted). First time participants in 2014 were more likely to use salt at the table than those participating for a second time (Odds Ratio (OR): 1.6 (1.1 to 2.4), but there was no difference in reported salt use during cooking. Participants living in regional areas (regional cities, large towns and surrounding agricultural areas) were less likely to add salt during cooking (OR: 0.5 (0.4, 0.7)).

Across both surveys, almost 60% reported sometimes or often/always adding salt at the table and sometimes or often/always adding salt in cooking (Table 2). Men were more likely than women to report adding salt at the table (OR: 1.5 (1.1, 2.0)), but not more likely to add salt in cooking (OR: 0.9 (0.7 to 1.2)). There was no association of age with reported salt use at the table. Categories of income were not associated with salt use at the table or in cooking, but those who were tertiary educated were marginally more likely to report adding salt when cooking (OR: 1.4 (1.0, 1.8)).

**Table 2:** Comparison of 24 h sodium and salt excretion (mean (95% CI) by frequency of use salt in cooking and at the table for individuals participating in both surveys (*n* = 733).

Reported Use of Salt	Frequency	%	Sodium mmol/Day ^a^ (95% CI)	Salt g/Day ^a^ (95% CI)	*p*-Value for Difference ^a,c^
					
Never/rarely add salt at the table	471	64%	133.3 (128.4, 138.0)	7.8 (7.5, 8.1)	0.052
Sometimes or often/always add salt at the table	262	36%	141.2 (134.8, 147.6)	8.3 (7.9, 8.6)	
Sometimes or often/always add salt when cooking ^b^	306	43%	139.7 (133.8, 145.7)	8.2 (7.8, 8.5)	0.073
Never/rarely add salt when cooking ^b^	409	57%	132.5 (127.4, 137.7)	7.7 (7.4, 8.0)	
Never/rarely add salt at the table & never/rarely add salt in cooking ^b^	296	41%	128.2 (122.2, 134.2)	7.5 (7.1, 7.8)	0.0016
Sometimes or often/always add salt at the table & sometimes or often/always add salt in cooking ^b^	419	59%	140.9 (135.8, 145.9)	8.2 (7.9, 8.5)	

^a^ gender adjusted; ^b^ 18 participants reporting that they do not cook were excluded; ^c^ Comparison of salt excretion among participants who “never/rarely add salt” *vs.* “sometimes or often/always add salt” conducted using post estimation procedures following ordinary least squares regression.

Mean daily salt excretion was 0.7 (0.3, 1.2) g/salt/day higher in participants reporting that they sometimes or often/always add salt at the table and when cooking, compared to those reporting never or rarely adding salt (Table 2). Participants reporting that they sometimes or often/always add salt at the table there was a 0.5 (−0.003, 0.9) (g/salt/day) higher daily salt intake compared to those who reported never or rarely adding salt at the table (*p* = 0.052). This trend for increasing 24 h salt excretion with increased frequency of discretionary salt use was evident for table salt (never add salt at the table: 7.7 (7.4, 8.0) g/salt/day compared to often and always add salt at the table: 8.6 (8.0, 9.2) g/salt/day (*p* = 0.001), although there was no significant difference between participants reporting sometimes add salt at the table 8.2 (7.7, 8.8) g/salt/day, compared to the group reporting “never” (*p* = 0.1).

However there was no significant a difference in daily salt excretion and reported use of salt in cooking alone, when comparing never add salt when cooking 7.7 (7.4, 8.0) g/salt/day to often/always add salt when cooking 8.2 (7.8, 8.5) g/salt/day (*p* > 0.07).

## 4. Discussion

The results of this study indicate that there has not been any significant reduction in population dietary sodium intake between 2011 and 2014 despite the establishment of the FHD in 2009 [25]. This failure to observe a reduction in sodium intake, using the objective measure of 24 h urinary excretion with a time frame of 3 years, is not surprising given the slow progress in reducing the sodium content of manufactured foods and no specific consumer salt awareness campaign. A number of assessments of the change in sodium content of categories of manufactured foods sold in Australia have been conducted and have found little change. Between 2008 and 2011 there was no reduction in sodium content of pasta sauces and average values were above the 2012 UK target [26]. During the same period there was no reduction in Australian ready meal products [27]. A more recent analysis does suggest a trend for some sodium reduction with an overall small fall in the mean sodium content of fast-food products between 2009 and 2012 [28] and between 2010 and 2013 there was a 9% fall in the mean sodium level in breads with almost 70% reaching the target together with a 25% reduction in the sodium content of breakfast cereal [26].

It should be noted that it took more than 7 years for the UK to demonstrate a significant fall in mean population salt intake (of 1.4 g/day between 2003 and 2011), assessed with 24 h urinary excretion [29]. The salt reduction initiatives in the UK that facilitated this significant reduction in population sodium intake were comprehensive and multi-faceted. The initiatives included government endorsement of a mean population salt intake target (6 g/salt/day); setting progressively lower salt targets for around 80 different categories of food, with clear time frames, assistance provided to industry to reformulate food with less salt and strong ministerial support; the promotion of clear nutritional labelling; delivery of consumer awareness campaigns with frequent surveys that generated media interest; and monitoring of progress with repeated 24-h urinary sodium at 3–5 years intervals, together with dietary reporting [29]. The first set of salt reduction targets for the food industry covered eighty-five food types in thirty different food categories and were designed to be met over a 4 years period [30]. In contrast the Australian FHD has moved very slowly, as over 6 years (2009–2015) only 9 food categories have sodium content target levels set. Furthermore a recent systematic interim assessment of FHD concluded that while the FHD’s goals were worthy, the mechanism for delivering was inadequate with little objective evidence of progress [31]. In contrast, in the UK between 2004 and 2011 salt levels in key food products were reduced including a 57% salt reduction in breakfast cereals, 30% reduction in soups, 25% in sweet biscuits and 24% in processed cheese and 20% in pies [29].

As a result of the salt reduction campaign conducted in the UK, 43% of adults in 2009 claimed to have made a special effort to reduce salt in their diet compared with 34% of adults in 2004, before the campaign commenced [30]. Furthermore the number of adults who generally added salt at the table fell from 32.5% in 2003 to 23.2% in 2007 [32] and the reduction in salt use at the table was significantly greater after the salt reduction campaign. This compares to our results with 37% reporting “sometimes/often/always” adding salt at the able in 2014 and 35% in 2011. Therefore the use of salt at the table in this population appears greater than that reported in the UK in 2003, although there are some differences in the wording of the question relating to frequency of use. Interestingly we did find that those participating for the first time in 2014 were more likely to use salt at the table than those participating for a second time which may indicate that participation in the study previously raised awareness and led to some moderation of discretionary use of salt, as all participants received their individual results with some educational messages. It is also likely that those participating in the two surveys were a particularly health conscious group. The reported frequency of table salt use in Australia tends to be higher with 60% reporting “sometimes/always” adding salt at the table [33] and another survey finding that 52% reported usually/always or sometimes adding salt at the table [34]. The most recent Australian health survey (2011) [35] found that a third of surveyed adults reported that they add salt “*very often*” during cooking or when preparing food and 16% of adults reported that they add salt “*very often*” at the table.

As there is evidence that consumers gradually adapt their taste to foods with less salt [36] and public education salt awareness campaigns do result in a reduction in table salt, a consumer education campaign would be worthwhile. This should be combined with a transparent accountable framework to facilitate the reduction of salt in manufactured foods, as it is likely that both of these changes contributed to the 15% reduction in population salt intake achieved in the UK [29].

Although dramatic population salt reductions are not possible, even relatively small reductions in mean population salt intake can contribute to improved health outcomes. The 15% reduction in population salt intake in the UK appears to have contributed to significant reductions in stroke (42%) and ischaemic heart disease (40%) during this period [37]. Therefore given it appears that no significant reduction in population sodium intake occurred during this 3 years time interval, it is imperative that government, public health agencies and manufacturers upscale the efforts to reduce the amount of sodium in the food supply. In May 2015, the Victorian Health Promotion Foundation launched its “State of Salt”, salt reduction initiative [38,39] with the ambitious goal of reducing the average daily salt intake of Victorian adults and children by 1 gram by 2018 with four intervention strategies. This initiative includes the development of voluntary programs with the food industry, increasing public awareness, strengthening existing policies and monitoring and evaluation. Although it is clear that progress can be made by voluntary initiatives between government and non-government agencies, there is a view that some legislation is required to limit the amount of salt added to the food supply. For example some countries such as South Africa have opted to legislate maximum levels of sodium in foods which may optimize progress [40]. Furthermore mandatory salt limits for processed food have been estimated to avert 18% of Australia’s burden of cardiovascular disease, which would be 20 times more than the health gains achieved by a voluntary reformulation program [41]. It would be worthwhile to give genuine consideration to a responsive regulatory model. It has been proposed that “legislative scaffolding” could be used to escalate from self-regulation towards co-regulation if industry fails to play its part in achieving national goals and targets. Utilizing a transparent framework to engage with the food industry’s wish to produce healthier foods could aid in speeding up the process of reformulation of foods with lower sodium content [42,43].

This evaluation of salt intake in a sample adult population residing in the state of Victoria during 2011 and 2014 clearly indicates that there was no reduction in salt intake as measured by 24 h urinary excretion of sodium. We had adequate power (80%, *p* < 0.05) to detect a mean change of 0.6 g/salt/day with 598 different participants in 2011 and 442 in 2014. Population changes in sodium intake, over time utilizing 24 h urine collections are usually conducted in different subjects [44] randomly approached, with only a limited number of those who are approached agreeing to provide 24 h collections (response rates ranging from 73% to 77%) [45]. It is not usual to follow up the same participants over time in studies assessing population sodium intake. Therefore, although 26% of participants in 2011 did not provide a 24 h urine collection in 2014, this did not negatively impact on our power to detect a meaningful change in sodium intake, as we recruited an additional 135 participants from the original larger population group which resulted in a sample size in 2014 sufficient to detect a 0.6 g/salt/day change in excretion with 80% power. The limitations of the study were that our sample population was not a representative population sample and included a lower proportion of participants less than 40 years, particularly in 2014. Residents from the most disadvantaged areas in the State, as determined by SEIFA were also under-represented in our sample, although there were similar proportions across both time points. The survey was conducted in only one state in Australia and therefore may not be representative of all urban and rural/regional areas, however as the bulk of manufactured foods and major brands are distributed throughout Australia it is likely that this sample population provides a reasonable estimation of salt excretion in the most densely populated areas. The average salt intake of 7.8 g/salt/day is likely to be an underestimate, as there was no adjustment for non-urinary losses. As those from lower socioeconomic backgrounds have been found to have higher dietary intakes of salt [46], the lower number of participants from lower SES areas in this sample suggests that our estimated mean salt intake may have underestimated population salt intake.

## 5. Conclusions

There is no indication that, over this 3-year period, initiatives such as the sodium targets set by the FHD in a limited number of food categories, resulted in a reduction in population salt intake in Australia, and there was no indication that consumers are reducing their use of discretionary salt which does contribute to daily salt intake. There is an urgent need to develop more effective strategies to reduce population salt intake, which should include increased engagement by government and industry, together with a consumer awareness campaign to reduce the use of discretionary salt.
